# The Mitogenome of the Haecon-5 Strain of *Haemonchus contortus* and a Comparative Analysis of Its Nucleotide Variation with Other Laboratory Strains

**DOI:** 10.3390/ijms25168765

**Published:** 2024-08-12

**Authors:** Yuanting Zheng, Neil D. Young, Jiangning Song, Robin B. Gasser

**Affiliations:** 1 Department of Biosciences, Melbourne Veterinary School, Faculty of Science, The University of Melbourne, Parkville, VIC 3010, Australia; yuantingz@student.unimelb.edu.au (Y.Z.); jiangning.song@monash.edu (J.S.); 2 Department of Data Science and AI, Faculty of IT, Monash University, Melbourne, VIC 3800, Australia; 3 Biomedicine Discovery Institute, Department of Biochemistry and Molecular Biology, Monash University, Clayton, VIC 3800, Australia; 4 Monash Data Futures Institute, Monash University, Clayton, VIC 3800, Australia

**Keywords:** *Haemonchus contortus*, laboratory strains, Haecon-5, mitochondrial genome, nucleotide variability

## Abstract

*Haemonchus contortus* (the barber’s pole worm)—a highly pathogenic gastric nematode of ruminants—causes significant economic losses in the livestock industry worldwide. *H. contortus* has become a valuable model organism for both fundamental and applied research (e.g., drug and vaccine discovery) because of the availability of well-defined laboratory strains (e.g., MHco3(ISE).N1 in the UK and Haecon-5 in Australia) and genomic, transcriptomic and proteomic data sets. Many recent investigations have relied heavily on the use of the chromosome-contiguous genome of MHco3(ISE).N1 in the absence of a genome for Haecon-5. However, there has been no genetic comparison of these and other strains to date. Here, we assembled and characterised the mitochondrial genome (14.1 kb) of Haecon-5 and compared it with that of MHco3(ISE).N1 and two other strains (i.e., McMaster and NZ_Hco_NP) from Australasia. We detected 276 synonymous and 25 non-synonymous single nucleotide polymorphisms (SNPs) within Haecon-5. Between the Haecon-5 and MHco3(ISE).N1 strains, we recorded 345 SNPs, 31 of which were non-synonymous and linked to fixed amino acid differences in seven protein-coding genes (*nad*5, *nad*6, *nad*1, *atp*6, *nad*2, *cyt*b and *nad*4) between these strains. Pronounced variation (344 and 435 SNPs) was seen between Haecon-5 and each of the other two strains from Australasia. The question remains as to what impact these mitogenomic mutations might have on the biology and physiology of *H. contortus*, which warrants exploration. The high degree of mitogenomic variability recorded here among these strains suggests that further work should be undertaken to assess the nature and extent of the nuclear genomic variation within *H. contortus*.

## 1. Introduction

*Haemonchus contortus*, commonly known as the barber’s pole worm, is a highly pathogenic gastric nematode of ruminants, such as sheep and goats. It causes significant economic losses in the livestock industry by affecting animal health and productivity [1,2]. The control of this and related parasites has relied heavily on the use of anthelmintics. However, the excessive use of these drugs has led to widespread drug resistance, making it increasingly challenging to manage infections and disease [3]. The extent of this resistance problem emphasises the need to develop novel drug targets and interventions to control *H. contortus* and related parasites effectively.

*H. contortus* has become a valuable model organism for anthelmintic discovery because well-defined laboratory strains, such as MHco3(ISE).N1 and Haecon-5, are available, because screening platforms have been developed, and because extensive genomic, transcriptomic and proteomic resources are accessible [4,5,6,7,8] for in-depth investigations. The Haecon-5 strain of *H. contortus* is used for anthelmintic screening and evaluation as well as for molecular studies that rely on the chromosome-contiguous genome available in MHco3(ISE).N1 for the purpose of analyses and annotation [6]. However, given previous evidence of genetic variability in mitochondrial DNA within *H. contortus* among relatively large numbers of adult male specimens (*n* = 223) from different geographical regions around the world [9], there is a need to genetically characterise Haecon-5 and explore the nature and extent of the genetic variability between the well-defined laboratory strains (i.e., MHco3(ISE).N1 and Haecon-5) routinely utilised for ongoing fundamental and applied investigations.

As a first step, here we (i) assembled and characterised the mitochondrial genome of the Haecon-5 strain; (ii) evaluated mitogenomic sequence variability within this strain; and (iii) genetically compared the mitogenomes of Haecon-5 with MHco3(ISE).N1 and/or other strains of *H. contortus*. At the outset, we hypothesised that there is no mitogenomic variability within the Haecon-5 population and only minor differences between Haecon-5 and other currently used laboratory strains.

## 2. Results

### 2.1. The Haecon-5 Mitogenome, and Variation within an Individual and a Population 

**Figure 1:**
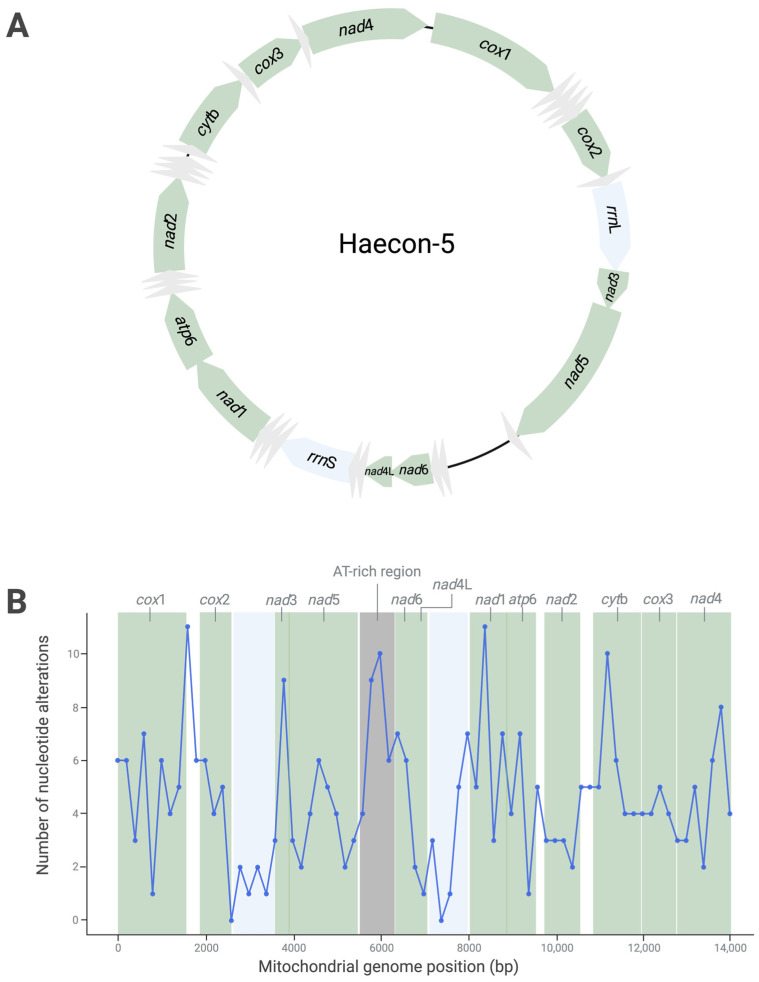
The circular mitochondrial genome of the Haecon-5 strain of *Haemonchus contortus* determined from a single adult male worm, with protein-coding (green), tRNA (grey) and rRNA (blue) genes, as well as non-coding regions (black), indicated (**A**). Nucleotide variability in the mitochondrial genome within a population of third-stage larvae (L3s; *n* = 200,000) of Haecon-5, established by a sliding window analysis of the linearised map (using a 200 bp window) (**B**). Table 2 details the nature and extent of this variability.

The complete mitochondrial genome of the Haecon-5 strain of *H. contortus* is 14,107 bp in length, has 36 genes (12 protein-coding genes, 22 transfer (t)RNA genes and 2 ribosomal (r)RNA genes) and is AT-rich (78%), consistent with the published mitogenomes of other strains (Figure 1A, Table 1).

Although there was no evidence of heteroplasmy within the single adult male of Haecon-5, marked variability (319 variants) was detected within the L3 population characterised by 301 (94.4%) SNPs, 15 (4.7%) indels and 3 (0.9%) MNPs (Figure 1; Table S1). Of all SNPs, 268 (89.1%) were transition (A↔G or C↔T) and 33 (10.9%) were transversion polymorphisms (A↔C, A↔T, G↔C or G↔T) (Figure 1; Table S1). Most SNPs (*n* = 238; 79%) were in the protein-coding genes (Figure 1), with 25 being non-synonymous and linked to amino acid alterations in *nad*5, *nad*6, *nad*1, *atp*6, *nad*2, *cyt*b and *nad*4 (Figure 1; Table 2). All indels were in non-coding regions, and MNPs resulted in amino acid alterations in the genes *nad*6 (ATA→GTT, leading to M→V) and *nad*2 (GT→AC, leading to V→T) (Table 2 and Table S1); small numbers of SNPs were located in non-coding regions (*n* = 63; 21.0%), tRNAs (*n* = 9) and rRNA regions (*n* = 9) (Figure 1). 

The sliding window analysis revealed an uneven distribution of nucleotide alterations across the genome (Figure 1B), with four main peaks. Three of these peaks were in protein-coding gene (*cox*1, *nad*1 and *cyt*b) regions. The fourth peak was in the AT-rich (“control”) region (Figure 1B). The highest number of SNPs, all of which were synonymous, were within the *cox*1 gene. In contrast, 13 alterations were recorded in *nad*6, 6 of which were non-synonymous—the most for any protein-coding gene (Table 2). As expected, variability in the AT-rich region was linked to 26 nucleotide alterations (Table 2 and Figure 1B).

### 2.2. Marked Mitogenomic Variability between Haecon-5 and Other Strains

**Figure 2:**
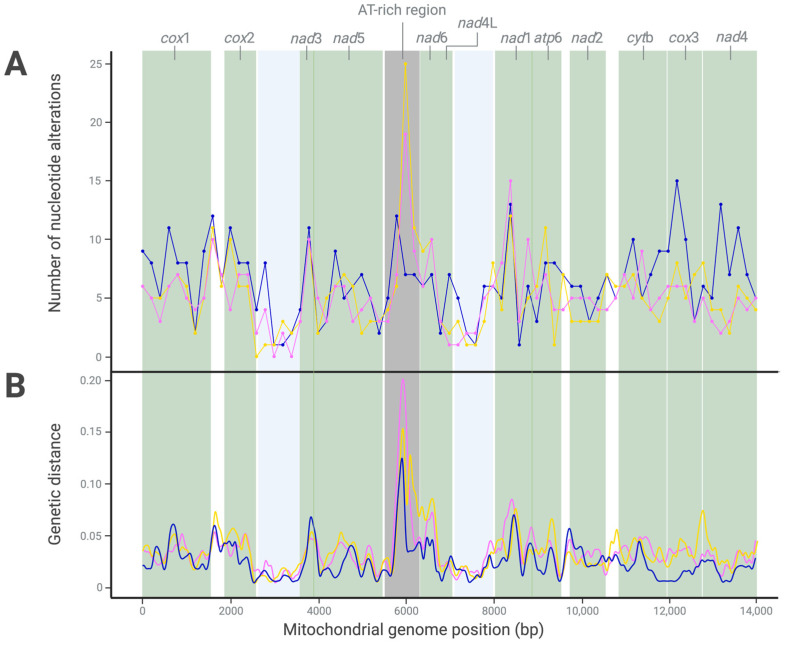
Nucleotide variability (**A**) and genetic distances (**B**) recorded upon pairwise comparison of the mitochondrial genomes of the Haecon-5 strain (Figure 1) with the MHco3(ISE).N1, McMaster and NZ_Hco_NP strains of *Haemonchus contortus* (pink, yellow and blue, respectively; refs. [6,10,11]) following a sliding window analysis (200 bp window). Table 3 details the nature and extent of this variability.

Pairwise mitogenomic comparisons of Haecon-5 with MHco3(ISE).N1, McMaster and NZ_Hco_NP revealed extensive nucleotide variability (Figure 2; Tables S2–S4). In total, 368 alterations were recorded between Haecon-5 and MHco3(ISE).N1, most (*n* = 278) of which were in coding regions, while 35 were linked to amino acid substitutions (Table 3). Most alterations (*n* = 345) were SNPs, and a minority were represented by indels (*n* = 16) and MNPs (*n* = 7) (Table 3). 

Similarly, the mitogenomic comparison between the Haecon-5 and McMaster strains revealed 378 alterations, including 283 in coding regions, 36 of which were non-synonymous (Table 3). The variants identified were classified as 344 SNPs, 14 MNPs and 20 indels (Figure 2). Most alterations (*n* = 460) were recorded between Haecon-5 and NZ_Hco_NP; 355 were located in coding regions and 30 were linked to amino acid alterations, almost half of which were in the genes *nad*1 and *nad*5 (Table 3). 

For all mitogenomic comparisons, the *cox*1 gene was the most variable, with 41 alterations in MHco3(ISE).N1, 42 in McMaster and 60 in NZ_Hco_NP. None of these alterations was linked to an amino acid change (Table 3). Conversely, the *nad*5 gene, which had the second highest number of nucleotide alterations, had the most non-synonymous SNPs (*n* = 6–8) (Table 3). The *nad*4L gene was least variable, with eight alterations—one of which was linked to an amino acid change (Table 3).

As expected, the AT-rich region (between 5531 and 6194 bp) was the most variable between Haecon-5 and each of the three other strains, reflected in the genetic distance results (Figure 2). Taken together, the variation was extensive among all four mitochondrial genomes, including 554 SNPs in coding regions, 63 of which were non-synonymous (Table 3 and Figure 2).

## 3. Discussion

Here, we characterised the mitochondrial genome of the Haecon-5 strain, assessed the mitogenomic sequence variability within this strain and compared the mitogenome of Haecon-5 with those of the strains MHco3(ISE).N1 from the UK, McMaster from Australia and NZ_Hco_NP from New Zealand. Based on our findings, we clearly rejected the hypotheses that (i) there is no mitogenomic sequence variability within the Haecon-5 population and that (ii) there are only minor differences between Haecon-5 and other currently used laboratory strains.

Although no heteroplasmy was detected within an individual adult male worm of Haecon-5, significant sequence variability was detected within the L3s population analysed—equating to 91.7% synonymous SNPs and 8.3% non-synonymous SNPs in all protein-coding genes. This magnitude of non-synonymous SNPs was unexpected and inferred to be associated with the amino acid changes at 27 positions in seven protein-coding genes. The nature and extent of the variability recorded (overall) supports a high mutation rate in this parasitic nematode [12,13,14] and explains the marked genetic diversity recorded in previous studies (e.g., [15,16,17]), particularly that of Sallé et al. [9], which revealed mitogenomic nucleotide sequence variation among 223 individual adult males of *H. contortus* from distinct countries and regions around the world. The results of the latter study were a major reason for assessing the extent of the genetic variability between laboratory strains, such as Haecon-5 and MHco3(ISE).N1, which are routinely employed for fundamental and applied investigations [6,18,19,20,21,22,23,24,25,26,27,28,29,30].

Thus, given that the laboratory strains MHco3(ISE).N1 and Haecon-5 strains have been, and will continue to be, used for decades for research purposes, we elected to directly compare these strains at the mitogenomic level. We also wanted to assess the extent of mitogenomic difference between these strains because the nucleotide genome of MHco3(ISE).N1 [6] has been used routinely as a “heterologous” reference for Haecon-5 for some years now without our knowing how genetically distinct it might be from Haecon-5. The results here (Section 2.2) indicated clearly that the MHco3(ISE).N1 and Haecon-5 strains are indeed unequivocally distinct (9.5% in the proteome-coding part of the mitogenome), which could have significant implications in relation to interpreting findings from molecular genetic investigations and conclusions made regarding, for example, the conservation of genes or proteins proposed as anthelmintic targets or vaccine molecules, or the functions of genes and their products.

Indeed, the marked genetic differences in the mitogenome (at 368 nucleotide positions) and mitoproteome (at 35 amino acid positions) between the strains Haecon-5 and MHco3(ISE).N1 of *H. contortus* suggest that some biological and/or phenotypic distinctiveness could exist. For instance, to date, there has been no direct comparison of the biology (e.g., life cycle–nematode development time and prepatency and patency periods), virulence or pathogenicity of these two strains. Moreover, no study has yet genetically characterised the distinct morphotypes of adult female worms (considering vulva types, cuticular ridge patterns and cervical papillae; refs. [31,32,33,34,35,36,37]) of these strains or examined the prevalence/abundance of these morphotypes in populations representing these strains. Indeed, it is possible that these morphotypes are genetically distinct genetic sub-types of *H. contortus* and that the dominant morphotype in each strain determines the mitogenome for each strain. This proposal remains to be explored. However, it is also possible that variation in the mitogenome sequences of *H. contortus* is not associated with distinct morphotypes but rather is the result of differences in development and/or mitonuclear coevolution (e.g., [38,39,40,41]).

## 4. Materials and Methods

The Haecon-5 strain of *H. contortus* was maintained using established protocols [26] approved by the University of Melbourne’s animal ethics committee (permit no. 23983). Genomic DNA was isolated from a single adult male worm (0.8 cm in length) or from a pool of 200,000 third-stage larvae (L3s) using the Circulomics tissue kit (Baltimore, MD, USA). The quality and quantity of DNA were assessed using the 4200 TapeStation (Agilent, Santa Clara, CA, USA). For short-read sequencing, genomic DNA from L3s or an adult male worm was used to construct paired-end libraries, which were sequenced (150 paired-end nucleotides) on the Illumina NextSeq500 or NOVAseq6000 platforms, and the data were stored in the FASTQ format. For long-read sequencing, libraries were prepared using SQK-LSK110 and SQK-LSK114 kits and sequenced on platforms from Oxford Nanopore Technologies (Oxford, UK), employing an established protocol [42]. Bases were called using Guppy v.6.4.6 from raw Pod5 files, with the output saved in FASTQ.

The mitochondrial genome of Haecon-5 was assembled from nanopore long-read sequences from a single male worm using Canu v.2.2 [43]. Protein-coding genes (PCGs), transfer RNAs (tRNAs) and ribosomal RNAs (rRNAs) were predicted using MITOS2 (http://mitos.bioinf.uni-leipzig.de/; accessed on 2 May 2024) and curated using Geneious Prime v.2024.0.5 [44]. The final annotated mitochondrial genome data were in FASTA and GFF3 formats for consistency and subsequent analyses.

For Haecon-5, short-read data representing a single adult male worm (used for assembly) or from pooled L3s were separately mapped and aligned independently to the homologous mitogenome using BWA v.0.7.17 [45] and sorted using SAMtools v.1.17 [46], and all duplicate reads were removed. Subsequently, single nucleotide polymorphisms (SNPs), insertion/deletion events (indels) and multi-nucleotide polymorphisms (MNPs) were explored using Geneious Prime v.2024.0.5 [44]. Each nucleotide alteration was recorded as synonymous or non-synonymous [47], with individual variants saved in the Variant Call Format (VCF).

Subsequently, the mitochondrial genomes of the MHco3(ISE).N1, McMaster and NZ_Hco_NP strains [6,10,11] and corresponding annotation files were downloaded from the National Center for Biotechnology Information (NCBI; https://www.ncbi.nlm.nih.gov/; accessed on 2 May 2024). Subsequently, a pairwise mitogenomic comparison of Haecon-5 with each of the three other strains was conducted (gene order and content, GC/AT composition and nucleotide variability), and nucleotide alterations (SNPs, indels and MNPs) were recorded using Geneious Prime v.2024.0.5, linked to a location in the mitogenome and annotated and saved in VCF. SPIDER v.1.1-2 [48] was used to calculate the genetic distance between Haecon-5 and the other strains. 

A sliding window analysis of aligned mitogenome sequences was conducted using GenomicRanges v.3.19 [49]. Alignment was achieved using MUSCLE v.5.1 [50] as implemented in Geneious Prime v.2024.0.5 [44]. A sliding window of 200 bases was used to estimate the nucleotide diversity (number of nucleotide alterations) and genetic distance between distinct strains of *H. contortus*. Nucleotide diversity was plotted against the midpoint of each window, and gene boundaries were defined.

## 5. Conclusions

The genetic distinctiveness between the strains MHco3(ISE).N1 and Haecon-5 of *H. contortus* at the mitogenomic level is pronounced. Given the utility of both of these strains for ongoing research in a wide range of areas, such as anthelmintic resistance, host–parasite interactions, drug discovery and vaccine development, the question remains as to how different these two strains are at the nuclear genomic and proteomic levels. This is the next question that we plan to address in the near future.

## Figures and Tables

**Table 1:** Features of the mitochondrial genomes of the Haecon-5 strain (determined herein), and of the MHco3(ISE).N1, McMaster and NZ_Hco_NP strains (published previously).

Features	Haecon-5 [This Study]	MHco3(ISE).N1 Ref. [6]	McMaster Ref. [10]	NZ_Hco_NP Ref. [11]
Mitochondrial genome size (bp)	14,107	14,018	14,055	14,001
Number of genes	36	36	36	36
Number of protein-coding genes	12	12	12	12
Number of tRNA genes	22	22	22	22
Number of rRNA genes	2	2	2	2
A (%)	33.0	33.2	33.4	32.9
C (%)	6.4	6.5	6.5	6.3
G (%)	15.6	15.5	15.4	14.8
T (%)	44.9	44.9	44.7	44.5
GC (%)	22.0	21.9	21.9	21.1
AT (%)	78.0	78.1	78.0	78.9
Predominant start codon	ATA	ATA	ATA	ATA
Predominant stop codon	TAA	TAA	TAA	TAA

A = adenine, C = cytosine, G = guanine and T = thymine.

**Table 2:** Nucleotide variation in the protein-coding, tRNA and rRNA genes, as well as the AT-rich (“control”) region of the mitochondrial genome, within a population of third-stage larvae (L3s) of the Haecon-5 strain of *Haemonchus contortus*.

Gene Name or Region	Description	Start	End	Length (bp)	Number of SNPs	Number of Indels	Number of MNPs	Total Number of Nucleotide Alterations
*cox*1	CDS	1	1582	1582	38[0]	0	0	38[0]
*cox*2	CDS	1861	2553	693	18[0]	0	1[0]	19[0]
*nad*3	CDS	3555	3881	327	6[0]	0	0	6[0]
*nad*5	CDS	3892	5473	1582	31[4]	0	0	31[4]
*nad*6	CDS	6362	6790	429	12[5]	0	1[1]	13[6]
*nad*4L	CDS	6797	7028	232	2[0]	0	0	2[0]
*nad*1	CDS	8044	8916	873	29[4]	0	0	29[4]
*atp*6	CDS	8918	9517	600	12[2]	0	0	12[2]
*nad*2	CDS	9704	10,549	846	14[3]	0	1[1]	15[4]
*cyt*b	CDS	10,816	11,928	1113	32[4]	0	0	32[4]
*cox*3	CDS	11,986	12,754	769	16[0]	0	0	16[0]
*nad*4	CDS	12,807	14,036	1230	28[3]	0	0	28[3]
*trn*C	tRNA	1583	1636	54	1	0	0	1
*trn*M	tRNA	1648	1704	57	0	0	0	0
*trn*D	tRNA	1728	1784	57	0	0	0	0
*trn*G	tRNA	1806	1860	55	1	0	0	1
*trn*H	tRNA	2554	2606	53	1	0	0	1
*trn*A	tRNA	5474	5530	57	0	0	0	0
*trn*P	tRNA	6195	6250	56	0	1	0	1
*trn*V	tRNA	6289	6343	55	1	0	0	1
*trn*W	tRNA	7029	7085	57	0	0	0	0
*trn*E	tRNA	7098	7152	55	0	0	0	0
*trn*S	tRNA	7853	7907	55	0	0	0	0
*trn*N	tRNA	7930	7985	56	1	0	0	1
*trn*Y	tRNA	7990	8043	54	2	0	0	2
*trn*K	tRNA	9532	9588	57	0	1	0	1
*trn*L	tRNA	9597	9651	55	0	0	0	0
*trn*S	tRNA	9652	9703	52	1	0	0	1
*trn*I	tRNA	10,561	10,619	59	0	0	0	0
*trn*R	tRNA	10,620	10,675	56	0	0	0	0
*trn*E	tRNA	10,676	10,730	55	1	0	0	1
*trn*F	tRNA	10,762	10,815	54	0	0	0	0
*trn*L	tRNA	11,929	11,985	57	0	2	0	2
*trn*T	tRNA	12,753	12,808	56	0	0	0	0
*rrn*L	rRNA	2607	3557	951	6	0	0	6
*rrn*S	rRNA	7151	7852	702	3	1	0	4
AT-rich region	non-coding	5531	6194	663	20	6	0	26

Note: Single nucleotide polymorphisms (SNPs); insertions and deletions (indels); multi-nucleotide polymorphisms (MNPs); protein-coding sequencing (CDS); transfer RNA gene (*trn*); small subunit ribosomal RNA gene (*rrn*S); large subunit ribosomal RNA gene (*rrn*L). The numbers of non-synonymous nucleotide alterations that lead to amino acid changes are indicated in square brackets.

**Table 3:** Nucleotide variability recorded upon pairwise comparison of the mitochondrial genomes of the Haecon-5 (this study) and other strains MHco3(ISE).N1, McMaster or NZ_Hco_NP; refs. [6,10,11]).

Comparisons	Number of		Protein-Coding Genes											
	SNPs; Indels; MNPs	Nucleotide Alterations (Total)	*cox*1	*cox*2	*nad*3	*nad*5	*nad*6	*nad*4L	*nad*1	*atp*6	*nad*2	*cyt*b	*cox*3	*nad*4
Haecon-5 vs. MHco3(ISE).N1	345[31]; 16[0]; 7[4]	368[35]	41[0]	22[0]	7[0]	37[6]	18[6]	3[0]	36[5]	16[5]	22[7]	32[3]	21[1]	23[2]
Haecon-5 vs. McMaster	344[31]; 20 (0); 14[5]	378[36]	42[0)	25[2]	7[0]	38[7]	23[5]	4[1]	28[5]	19[2]	15[4]	31[5]	22[2]	29[3]
Haecon-5 vs. NZ_Hco_NP	435[25]; 16[0]; 9[5]	460[30]	60[0]	29[1]	9[1]	44[8]	15[3]	4[0]	27[6]	19[3]	24[3]	38[2]	36[1]	50[2]
Among all strains	695[57]; 36[0]; 14[6]	745[63]	88[0]	43[3]	12[1]	71[12]	34[9]	8[1]	55[10]	37[6]	38[9]	59[5]	50[4]	59[3]

Note: Single nucleotide polymorphisms (SNPs); insertions and deletions (indels); multi-nucleotide polymorphisms (MNPs). The numbers of non-synonymous nucleotide alterations that lead to amino acid changes are indicated in square brackets.

## Data Availability

The complete mitochondrial genome assembly is available in the National Center for Biotechnology Information (NCBI) database under accession numbers PRJNA1071864 and PQ149937.

## Supplementary Materials

The supporting information can be downloaded at https://www.mdpi.com/article/10.3390/ijms25168765/s1.
